# Restaurant-Based Measures to Control Community Transmission of COVID-19, Hong Kong

**DOI:** 10.3201/eid2803.211015

**Published:** 2022-03

**Authors:** Faith Ho, Tim K. Tsang, Huizhi Gao, Jingyi Xiao, Eric H.Y. Lau, Jessica Y. Wong, Peng Wu, Gabriel M. Leung, Benjamin J. Cowling

**Affiliations:** World Health Organization Collaborating Centre for Infectious Disease Epidemiology and Control, University of Hong Kong, Hong Kong, China (F. Ho, T.K. Tsang, H. Gao, J. Xiao, E.H.Y. Lau, J.Y. Wong, P. Wu, G.M. Leung, B.J. Cowling);; Hong Kong Science and Technology Park, Hong Kong (E.H.Y. Lau, P. Wu, G.M. Leung, B.J. Cowling)

**Keywords:** COVID-19, coronavirus disease, SARS-CoV-2, severe acute respiratory syndrome coronavirus 2, influenza, viruses, respiratory infections, zoonoses, restaurant, community transmission, reproductive number, nonpharmaceutical interventions, Hong Kong, *Suggested citation for this article*: Ho F, Tsang TK, Gao H, Xiao J, Lau EHY, Wong JY, et al. Restaurant-based measures to control community transmission of COVID-19, Hong Kong. Emerg Infect Dis. 2022 Mar [*date cited*]. https://doi.org/10.3201/eid2803.211015

## Abstract

Controlling transmission in restaurants is an important component of public health and social measures for coronavirus disease. We examined the effects of restaurant measures in Hong Kong. Our findings indicate that shortening operating hours did not have an effect on time-varying effective reproduction number when capacity was already reduced.

As of April 14, 2021, a total of 11,608 cases and 207 deaths from coronavirus disease (COVID-19) had been reported in Hong Kong (*1*). A series of community epidemics have occurred, the largest of which have been the third wave in June–October 2020, which had 3,978 cases, and the fourth wave in November 2020–March 2021, which had 6,048 cases. To suppress local transmission of COVID-19, the government implemented a combination of public health and social measures (PHSMs): bar closures, restaurant capacity restrictions and opening hour restrictions, bans on live music performances and dancing, and work-from-home advisories (*2*). Ongoing assessment of the effect of these measures on transmission can guide evidence-based policy. One type of location in which COVID-19 transmission is known to occur is restaurants (*3*). Earlier studies have evaluated the impact of PHSMs, including restrictions on large group gatherings (*4*–*6*), but the specific effect of restaurant measures was not studied. Here we focus on the effect of restaurant measures on transmission in Hong Kong.

We collected details and time of implementation of each intervention of all the PHSMs applied during the third and fourth waves from the official reports of the Hong Kong government (*7*) (Appendix Table 1). In wave 3, a ban on dine-in service after 6:00 pm was in force during July 15–August 27, 2020 (Figure, panel A). Other PHSMs were implemented on the same day and kept in place for longer. Wave 4 was initiated by multiple superspreading events in a network of dancing venues. A ban on dine-in service after 6:00 pm was implemented on December 10, 2020, which was a week to a month later than the implementation of other PHSMs (Figure, panel B). Hence, we could disentangle the effect of shortened dine-in hours from other measures. No other PHSMs were implemented before the study period.

**Figure:**
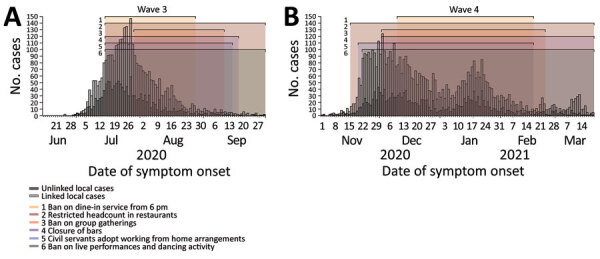
Use of public health and social measures (PHSMs) to reduce transmission of coronavirus disease in 2 waves of the epidemic, Hong Kong, 2020–2021. A) Incidence and implementation of PHSMs during wave 3, June 15–September 30, 2020. B) Incidence and implementation of PHSMs during wave 4, November 1, 2020–March 20, 2021. Dark and light gray bars represent the incidence of unlinked local cases and linked local cases of coronavirus disease in Hong Kong. Linked local cases are cases that are linked initially or after epidemiological investigation. Effective periods of PHSMs related to restaurants are shown in shaded areas in different colors.

To determine the effect of the ban on dine-in services after 6:00 pm, we applied a previous approach to estimate time-varying reproduction number (R_t_) (*8**,**9*). Then, we fitted LASSO regression models to log(R_t_) to assess the effect of the ban on dine-in services after 6:00 pm on R_t_, accounting for the effect from other PHSMs (*10*). We allowed for a 7-day lag between implementation of a measure and its effect on incidence, to account for the incubation period. In both waves, we grouped the PHSMs other than ban on dine-in services after 6:00 pm into a single variable to indicate the period when >3 of these other PHSMs were in place.

We estimated that the ban on dine-in services after 6:00 pm did not reduce R_t_ in both waves, but other PHSMs were associated with substantial reductions in R_t_. In wave 3, R_t_ rose rapidly to 4.5 on June 27, 2020, but ≈1 week after measures were applied it was <1.0 (Appendix Figure, panel A). Implementation of >3 other PHSMs was associated with a 53% (95% CI 44%–59%) decrease in R_t_ (Table).

In wave 4, R_t_ increased to 3.1 on November 16, 2020, and then decreased to ≈1.0 after PHSMs began (Appendix Figure, panel B). Implementation of >3 other PHSMs was associated with a 40% (95% CI 28%–47%) decrease in R_t_. Another model that excluded basic civil service arrangement in other PHSMs showed that a ban on dine-in service beginning at 6:00 pm did not have an effect (Table). We performed sensitivity analysis to remove the effect of superspreading in wave 3 by changing the start date to July 1, 2020; we found the ban on dine-in service from 6:00 pm did not have an effect (Appendix Table 2).

Our analysis suggested that the PHSMs were critical for suppressing the third and fourth waves of COVID-19 in Hong Kong. However, we found that a ban on dine-in hours after 6:00 pm might not have had an effect in both waves when capacity was already reduced. A complete closure of restaurants in Hong Kong would have considerable social impact because dining out is very common. We hypothesize that encouraging restaurants to extend dine-in hours, but with capacity restrictions to reduce crowding, could be a reasonable approach to reduce transmission. 

A limitation of our analysis is that we cannot distinguish the effect of some PHSMs because they began simultaneously. We cannot rule out that a ban on dine-in service after 6:00 pm might have an effect if it began earlier than other PHSMs or in regions with high incidences. In addition, changes in R_t_ are a consequence of individual behavioral changes such as avoiding crowded areas; increasing incidence and implementation of multiple PHSMs could raise the public’s perception of risk. Determining the effectiveness of alternative PHSMs would provide evidence-based guidance on control strategies.

**Table:** 

**Table.** Effect on time-varying reproduction number of public health and social measures in waves 3 and 4 of COVID-19, Hong Kong, 2020–2021


**Table:** 

PHSM	% Change in R_t_ (95% CI)
Model 1	
Wave 3	
Ban on dine-in service after 6:00 pm†	0
>3 other PHSMs‡	−53 (−59 to −44)
Wave 4	
Ban on dine-in service after 6:00 pm	0
>3 other PHSMs	−40 (−47 to −28)
Model 2	
Wave 3	
Ban on dine-in service after 6:00 pm	0
>3 other PHSMs, excluding basic civil service arrangement	−51 (−57 to −43)
Wave 4	
Ban on dine-in service after 6:00 pm	0
>3 other PHSMs, excluding basic civil service arrangement	−38 (−46 to −27)

*Wave 3 was June 15–September 30, 2020; wave 4 was November 1, 2020–March 15, 2021. COVID-19, coronavirus disease; PHSM, public health and social measure; R_t_, reproduction number. †Because of variable selection and regularization in LASSO regression, the regression coefficient was shrunk to 0 in the model. ‡Other PHSMs include restricted headcount in restaurants, ban on group gatherings, bar closure, flexible civil service arrangement, and ban on live performances and dancing activity.

Appendix Additional information about public health and social measures to reduce transmission of coronavirus disease in 2 waves of the epidemic, Hong Kong, 2020–2021
