# Supplementary material for: Restaurant-Based Measures to Control Community Transmission of COVID-19, Hong Kong
Source: Emerg Infect Dis. 2022 Mar;28(3):759–61. doi: 10.3201/eid2803.211015 (PMC8888230; doi:10.3201/eid2803.211015)
Supplement: Appendix — Additional information about public health and social measures to reduce transmission of coronavirus disease in 2 waves of the epidemic, Hong Kong, 2020–2021 [file 21-1015-Techapp-s1.pdf]

Article DOI: https://doi.org/10.3201/eid2803.211015

Restaurant-Based Measures to Control Community Transmission of COVID-19, Hong Kong

Appendix

Additional Methods

We used LASSO time-series regression to compute the association of restaurant measures and reduced transmissibility ($log(R_t)$). Ban on dine-in service after 6:00 P.M. was treated as one dummy variable. Restricted headcount in restaurants, ban on group gatherings, closure of bars, flexible working arrangements, and ban on live performances and dancing activity were grouped as a second dummy variable to indicate whether ≥3 other public health and social measures (PHSMs) were implemented on any day.

In model 1, x_{ij} includes ban on dine-in service from 6:00 P.M. in waves 3 (June 15–September 30, 2020) and 4 (November 1, 2020–March 20, 2021), and ≥3 other PHSMs in waves 3 and 4. In model 2, x_{ij} includes ban on dine-in service from 6:00 PM in waves 3 and 4 and also ≥3 other PHSMs, excluding basic civil service arrangement, in waves 3 and 4.

Mathematically, the equation for model 1 is

$$Log(R_t) = \beta_1 * Ban\ on\ dinein\ service\ after\ 6:00\ \text{PM}\ in\ Wave\ 3 + \beta_2 * \geq 3\ other\ PHSMs\ implemented\ in\ Wave\ 3 + \beta_3 * Ban\ on\ dinein\ service\ after\ 6:00\ \text{PM}\ in\ Wave\ 4 + \beta_4 * \geq 3\ other\ PHSMs\ implemented\ in\ Wave\ 4$$

The equation for Model 2 is

$$Log(R_t) = \beta_1 * Ban\ on\ dinein\ service\ after\ 6:00\ \text{P.M.}\ in\ Wave\ 3 + \beta_2 * \geq 3\ other\ PHSMs\ implemented\ excluding\ basic\ service\ arrangement\ in\ Wave\ 3 + \beta_3 * Ban\ on\ dinein\ service\ after\ 6:00\ \text{PM}\ in\ wave\ 4 + \beta_4 * \geq 3\ other\ PHSMs\ implemented\ excluding\ basic\ service\ arrangement\ in\ Wave\ 4$$

The likelihood is as follows:

$$L_{lasso}(\hat{\beta}) = \sum_{t=1}^{n} (log(R_t) - \sum_{j} x_{tj}\beta_j)^2 + \lambda \sum_{j=1}^{p} |\beta_j|$$

λ is the amount of shrinkage and was determined within 1 standard error from the minimum by cross-validation.

All analysis was conducted in R version 3.6.1 (http://www.r-project.org).

Appendix Table 1. Public health and social measures included in analysis of their impact in control of coronavirus transmission*

		Effective period	
PHSM	Details	Wave 3	Wave 4
(1) Ban on dine-in service from 6:00 PM	Catering premises must stop selling or supplying food or drink for consumption on their premises from 6:00 PM to 4:59 AM the subsequent day.	Jul 15–Aug 27	Dec 10–Feb 17
(2) Restricted headcount in restaurants	No more than 2 (or 4) people can sit together at one table within any catering premises and not more than 50% of their seating capacity is allowed.	Jul 15–Oct 29	Nov 16 to date
(3) Ban on group gatherings	A group gathering of more than 2 people in public places is prohibited.	Jul 29–Sep 10	Dec 2–Feb 23
(4) Closure of bars	All bars, pubs and nightclubs must be closed.	Jul 15–Sep 17	Dec 2 to date
(5) Civil servants adopt working from home (WFH) arrangements	Basic service arrangement: All government employees should have flexible work schedule provided that limited public services would be maintained.	Jul 15–19; Aug 24–Sep 14	Nov 20–Dec 1; Jan 28–Feb 17
	Minimal service arrangement: Apart from those departments providing emergency and essential public services, all government employees should work from home.	Jul 20–Aug 23	Dec 2–Jan 27
(6) Ban on live performances and dancing activity	Live performances and dancing activity are not allowed in catering premises (and clubhouses in Wave 4).	Jul 15–Oct 29	Nov 22 to date

*Data were current to March 31, 2021.PHSM, public health and social measures.

Appendix Table 2. Sensitivity analysis of impact on time-varying reproduction number of public health and social measures in waves 3 and 4 of coronavirus disease, Hong Kong, 2020–2021*

PHSM	% Change in R_t (95% CI)
Model 1	
Wave 3	
Ban on dine-in service from 6:00 PM†	0
≥3 other PHSMs‡	−47 (−54 to −37)
Wave 4	
Ban on dine-in service from 6:00 PM†	0
≥3 other PHSMs	−33 (−42 to −19)
Model 2	
Wave 3	
Ban on dine-in service from 6:00 PM†	0
≥3 other PHSMs, excluding basic civil service arrangement	−44 (−51 to −34)
Wave 4	
Ban on dine-in service from 6:00 PM†	0
≥3 other PHSMs, excluding basic civil service arrangement	−29 (−39 to −17)

*Wave 3 was June 15–September 30, 2020; wave 4 was November 1, 2020–March 20, 2021. COVID-19, coronavirus disease; PHSM, public health and social measure; R_t, reproduction number.
†Due to variable selection and regularization in LASSO regression, regression coefficient was shrunk to 0 in the model.
‡Other PHSMs include restricted headcount in restaurants, ban on group gatherings, bar closure, flexible civil service arrangement, and ban on live performances and dancing activity.

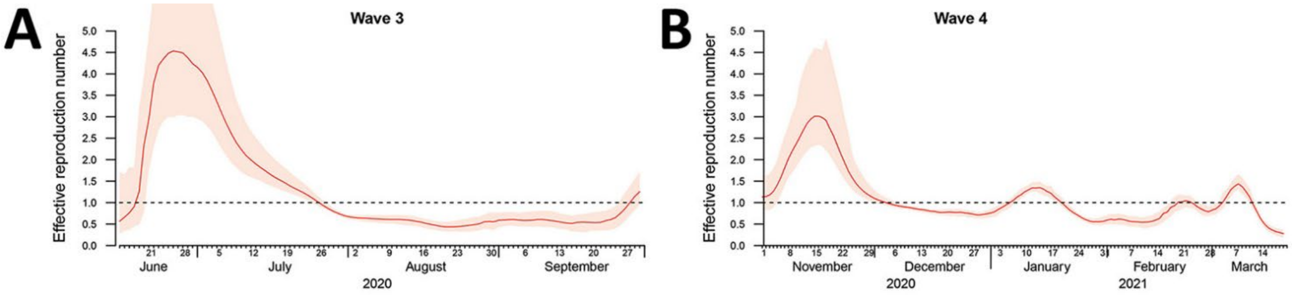

Appendix Figure. Transmissibility of coronavirus disease (COVID-19) in 2 waves of epidemic, Hong Kong, 2020-2021. A) Effective reproduction number (R_t) during wave 3, June 15–September 30, 2020. The red line and pink shaded area represent the estimated R_t and 95% CIs respectively. The dotted line indicates the critical threshold of R_t=1. B) Effective reproduction number during wave 4, November 1, 2020–March 20, 2021.
